# Risk-factors for re-admission and outcome of patients hospitalized with confirmed COVID-19

**DOI:** 10.1038/s41598-021-96716-7

**Published:** 2021-08-31

**Authors:** Hefziba Green, Dafna Yahav, Noa Eliakim-Raz, Nitzan Karny-Epstein, Shiri Kushnir, Tzippy Shochat, Boaz Tadmor, Alon Grossman

**Affiliations:** 1 Department of Medicine B, Rabin Medical Center, Beilinson Campus, Jabutisky St 39, 4941492 Petah Tiqva, Israel; 2 Sackler Faculty of Medicine, Tel-Aviv University, Tel Aviv, Israel; 3 Infectious Diseases Unit, Rabin Medical Center, Beilinson Campus, Petah Tiqva, Israel; 4 Department of Medicine E, Rabin Medical Center, Beilinson Campus, Petah Tiqva, Israel; 5 Research Authority, Rabin Medical Center, Beilinson Campus, Petah Tiqva, Israel; 6 Bio-statistics Unit, Rabin Medical Center, Beilinson Campus, Petah Tiqva, Israel

**Keywords:** Epidemiology, Outcomes research

## Abstract

Burden of COVID-19 on Hospitals across the globe is enormous and has clinical and economic implications. In this retrospective study including consecutive adult patients with confirmed SARS-CoV-2 who were admitted between 3/2020 and 30/9/20, we aimed to identify post-discharge outcomes and risk factors for re-admission among COVID-19 hospitalized patients. Mortality and re-admissions were documented for a median post discharge follow up of 59 days (interquartile range 28,161). Univariate and multivariate analyses of risk factors for re-admission were performed. Overall, 618 hospitalized COVID-19 patients were included. Of the 544 patient who were discharged, 10 patients (1.83%) died following discharge and 50 patients (9.2%) were re-admitted. Median time to re-admission was 7 days (interquartile range 3, 24). Oxygen saturation or treatment prior to discharge were not associated with re-admissions. Risk factors for re-admission in multivariate analysis included solid organ transplantation (hazard ratio [HR] 3.37, 95% confidence interval [CI] 2.73–7.5, *p* = 0.0028) and higher Charlson comorbidity index (HR 1.34, 95% CI 1.23–1.46, *p* < 0.0001). Mean age of post discharge mortality cases was 85.0 (SD 9.98), 80% of them had cognitive decline or needed help in ADL at baseline. In conclusion, re-admission rates of hospitalized COVID-19 are fairly moderate. Predictors of re-admission are non-modifiable, including baseline comorbidities, rather than COVID-19 severity or treatment.

## Introduction

At the end of 2019 the SARS-CoV-2 has emerged as a pandemic causing corona virus disease 2019 (COVID-19). COVID-19s’ impact on hospital burden is enormous and has clinical and economic consequences^1,2^. In the early outbreak, admission length of stay was relatively long especially in China, with median length of stay of 14 days compared to 5 in other countries^3^. In the following months overall length of stay for patients who did not require intensive care unit (ICU) decreased, but is still 5–7 days^4,5^. Discharge and post-discharge planning of patients hospitalized with COVID-19 may help the strategy of reducing the high burden of admissions. Identifying patients who can be safely discharged and the parameters that predict re-admission is essential for this purpose. While data on the risk factors for re-admission is accumulated, the clinical course of patients with COVID-19 following discharge and upon re-admission is less well described^4–12^. For this purpose, we sought to examine which parameters are associated with worse outcome or re-admission of discharged patients.

## Methods

This was a retrospective study, including consecutive adult (> 18 years) patients with confirmed SARS-CoV-2 (see definitions below) who were admitted to one of the two hospitals of Rabin medical center. Rabin medical center is a tertiary center, which consists of two separate hospitals with a total capacity of 1300 beds. Beginning in March 2020, four medical wards and two intensive care units were adjusted for the treatment of patients with COVID-19. The end of follow-up was defined as 1/10/20. Patients who were still hospitalized at the end of follow-up or died in the index admission were excluded from the analysis. Data were collected from medical electronic files and were available for all patients. Data were cross-referenced with the Israel National death registry to identify patients who died following discharge from the index admission. Data on re-admission was obtained from the local center computerized medical files, as well as from a nationwide electronic medical records that contains information about hospitalizations nationwide. Re-admission was defined as the first admission for any cause at any ward to any hospital in Israel following discharge from the index admission with COVID-19.

Patients hospitalized with confirmed COVID-19 are discharged to one of the following places: homes, with continuing care of home referral team (telemedicine), taking into considerations isolation options; community centers adjusted health facilities for independent patients who have difficulties staying home; specialized long term care facilities adjusted for elderly, and/or needing-assistance patients with COVID-19 (skilled nursing homes).

Confirmed SARS-CoV-2 was defined as a positive respiratory sample for RT-PCR for SARS-CoV-2, using commercial RT-PCR methods (either Seegene AllplexTM2019-nCoV Assay (Seegene, Seoul, South Korea) or GeneXpert). COVID-19 disease severity was defined based on the National Institute of Health (NIH) guidelines, which were adopted by the Israel Ministry of Health^13^. Definition of recovery was based on CDC recommendations^14^.

Treatment for COVID-19 during hospitalization has evolved during the time period, and was updated periodically based on the NIH, WHO and Israeli guidelines^13,15,16^. During the early outbreak, hydroxychloroquine and azithromycin were administrated as part of the initial therapy, but this practice was later abandoned. More recent guidelines were adopted for hospitalized patients: for patients with severe or critical disease requiring oxygen supplementation remdesivir (loading dose of 200 mg and then 100 md/day for overall 5 days or until discharge, which ever was earlier) and dexamethasone, in a dose of 6 mg/day for 10 days or until discharge (whichever was earlier). All other therapies, such as convalescent plasma, anti IL-1 or IL-6 where dispensed only as part of clinical trials.

The variables evaluated as potential risk factors for re-admission were chosen based on their relevance to COVID-19 severity and outcome according to the current literature^8,17–19^. The majority of them are also considered risk factors for re-admission in the general population^20,21^. Age was recorded both as continuous variable and classified into age groups (18–29, 30–39 etc. until 80+). Comorbidities included the individual components of the Charlson Comorbidity Index (CCI) score^22^: severe kidney disease, coronary artery disease (CAD), congestive heart failure (CHF), peripheral vascular disease (PVD), diabetes mellitus (DM) and DM target organ damage, leukemia, lymphoma, cerebrovascular disease, chronic liver disease, metastatic disease, chronic pulmonary disease, dementia, AIDS and peptic ulcer disease. Total CCI score was also calculated. Other demographic variable included gender, weight, solid organ transplant status, and baseline functional capacity.

Data on index admission included hospital length of stay, recorded as a continuous variable, and also classified into 3 groups: less than 24 h, 1–7 days and more than 7 days; oxygen saturation (SpO2, minimum and last value before discharge, measured with or without oxygen supplementation), temperature, systolic and diastolic blood pressure before discharge and the administration of dexamethasone and/or remdesivir. Laboratory tests were recorded on index admission first day and before discharge, including total white blood cells and lymphocyte count, hemoglobin level, platelets, serum creatinine, sodium and potassium, total bilirubin and inflammatory markers (CRP, ferritin, fibrinogen, D-dimer).

The study was approved by the Rabin Medical Center review board and conducted in accordance with the declaration of Helsinki. All experimental protocols were approved by the Rabin Medical Center licensing committee. Due to the retrospective design of the study, and since all data was retrieved and kept anonymously, informed consent was waived by the Rabin Medical Center local ethical committee (approval number 0893-20-RMC).

### Statistical analysis

The statistical analysis for this paper was generated using SAS Software, Version 9.4. Continuous variables were presented by Mean (SD), Categorical variables were presented by (N, %). Hazard ratios for readmission were computed using the Cox proportional hazards model; with the Fine and Gray methodology of correcting for the competing risk of death, with no readmission. The Fine and Gray methodology is used in the Cox proportional hazards model, for time till re-admission, where death without readmission is a competing risk (patients who died, without readmission, have a zero probability of future readmission, and as such, cannot be censored)^23^. No variable selection was done. Variables included in the model were chosen by clinical reasoning, based on data from previous studies regarding outcome and prognosis of patients with COVID-19. The above Cox model was also used to perform a multiple regression model, to find a minimal set of factors that are related to readmission.

## Results

Overall, 618 patients were hospitalized with confirmed COVID-19 during the study period. Seventy-one died during the index admission and were not included in the analysis. Of the 544 patient who were discharged, 10 patients (1.83%) died following discharge and were included in the competing risk analysis and 50 patients (9.2%) were re-admitted. Median post discharge follow-up was 59 days (Interquartile range, IQR, 28-161). Characteristics of patients re-admitted compared with those not re-admitted are presented in Table 1. Mean age of patients who were re-admitted was 68.04 (16.98) years versus 57.17 (18.39) years of those who were not re-admitted. Slightly more than half were male. Median duration of the index admission was 5 days in both groups (IQR 2–11 days versus 3–10 days in the re-admitted versus nor-readmitted groups, respectively). Patients who were re-admitted had more comorbidities, as reflected in the significantly higher total CCI; 4.22 (2.74) versus 2.19 (2.03).

**Table 1:** Patients’ characteristics.

	Alive, no re-admission (N = 484)	Readmission (N = 50)	Dead, no readmission (N = 10)	Total (N = 544)
Age, mean (SD)	57.17 (18.39)	68.04 (16.98)	85.0 (9.39)	58.68 (18.74)
**Age_group**				
18–29	46 (9.5%)	2 (4%)	0 (0%)	48 (8.8%)
30–39	47 (9.7%)	0 (0%)	0 (0%)	47 (8.6%)
40–49	67 (13.8%)	6 (12%)	0 (0%)	73 (13.3%)
50–59	83 (17%)	5 (10%)	0 (0%)	88 (16.1%)
60–69	108 (22.3%)	10 (20%)	0 (0%)	118 (21.6%)
70–79	79 (16.3%)	13 (26%)	3 (30%)	95 (17.4%)
80+	54 (11.6%)	714 (28%)	7 (70%)	75 (13.79%)
Female (N, %)	235 (48.5%)	18 (36%)	4 (40%)	257 (47.2%)
**Duration of admission**				
< 24 h	44 (9.0%)	6 (12%)	0 (0%)	50 (9.3%)
1–7 d	297 (61.3%)	28 (56%)	7 (70%)	332 (61.1%)
> 7 d	143 (29.5%)	16 (32%)	3 (30%)	162 (29.7%)
**Baseline functional capacity**				
Independent	366 (75.6%)	28 (56%)	2 (20%)	396 (72.8%)
Needs assistance in ADL	35 (7.2%)	11 (22%)	7(70%)	53 (9.7%)
Cognitive impairment	33 (6.82%)	10 (20%)	1 (10%)	44 (8.2%)
Weight, Kg, mean (SD) N = 443	80.77 (17)	78.98 (16.99)	68.50 (16.1)	80.30 (17.04)
Total admission days (median, 25–75%)	5.00 (3–10)	5.5 (2–11)	5.50 (3–11)	5.00 (3–10)
Charlson comorbidity index (CCI), mean (SD)	2.19 (2.03)	4.22 (2.74)	5.2 (2.2)	2.44 (2.22)
**Comorbidities (Charlson, %)**				
Severe kidney disease	9 (1.8%)	3 (6%)	0 (0%)	12 (2.2%)
Coronary artery disease	32 (6.61%)	9 (18%)	1 (10%)	42 (7.2%)
Congestive heart failure	21 (4.34%)	5 (10%)	2 (20%)	28 (5.1%)
Peripheral vascular disease	0 (0%)	0 (0%)	0 (0%)	0 (0%)
Diabetes mellitus	81 (16.7%)	18 (36%)	2 (20%)	101 (18.7%)
Diabetic retinopathy/neuropathy/nephropathy	3 (0.6%)	1 (2.0%)	0 (0%)	4 (0.7%)
Leukemia	2 (0.4%)	0 (0%)	0 (0%)	2 (0.37%)
Lymphoma	4 (0.83%)	1 (2%)	0 (0%)	5 (0.9%)
Cerebrovascular disease	30 (6.2%)	8 (16%)	4 (40%)	42 (7.7%)
Hemiplegia	0 (0%)	0 (0%)	0 (0%)	0 (0%)
Metastatic solid tumor	14 (2.89%)	8 (16%)	1 (10%)	23 (4.23%)
Dementia	1 (0.2%)	1 (2%)	0 (0%)	2 (0.4%)
Chronic pulmonary disease	33 (6.8%)	6 (12%)	2 (20%)	41 (7.5%)
Connective tissue disease	11 (2.27%)	3 (6%)	0 (0%)	14 (2.6%)
Peptic ulcer disease	11 (2.27%)	5 (10%)	1 (10%)	17 (3.1%)
AIDS	1 (0.2%)	0 (0%)	0 (0%)	1 (0.18%)
Liver disease	6 (1.2%)	2 (4%)	0 (0%)	8 (1.5%)
Solid organ transplantation	8 (1.65%)	5 (10%)	0 (0%)	13 (2.4%)
**Type—Solid organ transplantation**				
Kidney	7 (1.45%)	3 (6%)	0 (0%)	10 (1.8%)
Heart	1 (0.2%)	1 (2.0%)	0 (0%)	2 (0.4%)
Liver	0 (0%)	1 (2.0%)	0 (0%)	1 (0.2%)
Highest temperature 48 h before discharge, mean (SD), N = 528	37.12 (0.59)	37.21 (0.63)	37.7 (0.72)	37.14 (0.61)
Last SpO2 48 h before discharge, mean (SD) N = 513	96.87 (2.57)	96.23 (3.10)	94.78 (2.05)	96.77 (2.63)
Minimum SpO2 48 h before discharge, mean (SD) N = 513	95 (3.38)	94.35 (4.45)	91.67 (4.74)	94.88 (3.54)
Systolic blood pressure 48 h before discharge, mean (SD) mmHG, N = 509	127.76 (17.73)	133.25 (16.07)	133.11 (15.06)	128.33 (17.6)
Diastolic blood pressure 48 h before discharge, mean (SD) mmHG, N = 509	72.0 (10.33)	71.75 (15.12)	72.18 (13.34)	71.99 (10.85)
Dexamethasone	113 (23.35%)	9 (18%)	2 (20%)	124 (22.7%)
Remdesivir	50 (10%)	3 (6%)	0 (0%)	53 (9.7%)
WBC on admission, mean (SD) N = 504	6.64 (3.15)	6.43 (3.27)	11.29 (8.8)	6.71 (3.4)
Lymphocytes on admission, mean (SD) N = 504	1.24 (0.89)	1.27 (0.75)	3.31 (5.42)	1.28 (1.17)
Hemoglobin on admission, mean (SD) N = 504	12.89 (1.75)	12.44 (2.46)	11.34 (1.64)	12.81 (1.84)
Platelets on admission, mean (SD) N = 504	225.53 (88.37)	212.92 (91.08)	246.8 (93.84)	224.65 (88.62)
C reactive protein on admission (median, 25–75%, N = 460)	4. 8 (1.25, 10.24)	2.7 (1.1, 5.68)	2.2 (1.57, 6.66)	4.28 (1.21, 9.64)
Fibrinogen on admission, mean (SD) N = 236	416.09 (98.85)	355.26 (59.42)	357.67 (28.1)	410.45 (97.18)
D-dimer on admission (median, 25–75%, N = 415)	710.5 (429, 1249)	823.00 (390, 1729)	834 (423, 3430)	713.00 (423, 1266)
Ferritin on admission (median, 25–75%, N = 280)	380.4 (174.6, 726.8)	170.6 (109.2, 445.1)	141.2 (83.8, 230.2)	356.90 (158.5, 717.3)
Creatinine on admission (median, 25–75%, mg/dl, N = 491)	0.88 (0.7, 1.1)	0.95 (0.77, 1.27)	1.02 (0.72, 1.2)	0.89 (0.7, 1.11)
Bilirubin on admission, mean (SD), mg/dl, N = 483	0.48 (0.3)	0.44 (0.27)	0.46 (0.25)	0.48 (0.3)
Sodium on admission, mean (SD), mg/dl, N = 494	137.52 (4.38)	137.21 (5.18)	137.6 (5.25)	137.49 (4.47)
Potassium on admission, mean (SD) mg/dl, N = 494	4.13 (0.58)	4.27 (0.64)	4.55 (0.61)	4.15 (0.59)
WBC on discharge, mean (SD) N = 504	7.0 (3.21)	6.18 (2.65)	8.53 (5.66)	6.96 (3.23)
Lymphocytes on discharge, mean (SD) N = 504	1.47 (0.92)	1.18 (0.69)	2.73 (4.07)	1.46 (1.06)
Hemoglobin on discharge, mean (SD) N = 504	12.45 (1.99)	12.24 (2.77)	11.27 (1.78)	12.41 (2.06)
Platelets on discharge, mean (SD) N = 504	270.25 (128.72)	243.67 (119.59)	239.9 (103.15)	267.38 (127.34)
C reactive protein on discharge (median, 25–75%, N = 462)	3.05 (0.95, 6.89)	2.01 (0.6, 5.31)	6.19 (1.57, 9.4)	2.91 (0.93, 6.78)
Fibrinogen on discharge, mean (SD) N = 236	430.4 (101.88)	357.53 (87.1)	316.3 (79.03)	398.64 (101.42)
D_dimer on discharge (median, 25–75%, N = 415)	746 (475, 1279)	884 (502, 1564)	2856 (423, 4678)	762 (475, 1316)
Ferritin on discharge (median, 25–75%, N = 280)	349.4(162.9, 652.6)	180.6 (121.3, 523.5)	286.25 (162.5, 445.2)	339.80 (158.9, 622.2)
Creatinine on discharge (median, 25–75%, N = 491)	0.8 (0.64, 0.99)	0.82 (0.7, 1.03)	0.89 (0.71, 1.04)	0.81 (0.65, 0.99)
Bilirubin on discharge, mean (SD) N = 483	0.45 (0.25)	0.44 (0.22)	0.42 (0.23)	0.45 (0.24)
Sodium on discharge, mean (SD) N = 494	138.11 (3.26)	138.15 (3. 76)	137.7 (6.68)	138.1 (3.4)
Potassium on discharge, mean (SD) N = 494	4.29 (0.47)	4.23 (0.45)	4.47 (0.39)	4.29 (0.47)

Among re-admitted patients, median time to re-admission was 7 days (IQR 3, 24). Eighteen (36%) patients, 11 (61%) of them within the first week after discharge, were re-admitted for severe respiratory symptoms related to COVID-19. Twelve were hospitalized with respiratory symptoms; five required invasive mechanical ventilation on admission; all classified as having severe COVID-19 disease although differentiation from bacterial superinfection was not certain. Six (50%) of the patients who were re-admitted with severe COVID-19 died during re-admission. Causes for re-admission and patients’ outcome are described in Table 2. Seventeen patients (34%) were considered recovered from COVID-19 upon readmission.

**Table 2:** Clinical characteristics of re-admitted patients.

COVID status^13,14^ on readmission	Reason for re-admission	N	Age, mean (SD)	Gender (male, N%)	Time from discharge to re-admission (days, mean (SD)	Outcome of readmission
Critical	Respiratory failure, mechanical ventilation	5	81.6 (7.3)	4 (80%)	5.4 (6.25)	4 died 1 discharge
Severe	Respiratory symptoms (including dyspnea, chest pain, pneumonia)	13	63.8 (14.4)	10 (76%)	4.53 (4.5)	2 died 11 discharged
Moderate (N = 6)			75 (12.03)	1 (16%)	4.8 (2.7)	All discharged
	Fever	2				
	Congestive heart failure	1				
	Other (hemolytic anemia, nausea, otitis)	3				
Mild (N = 9)			66.3 (19.11)	7 (77%)	3.6 (3.1)	All discharged
	Fever, weakness	3				
	CVA	1				
	Other (vertigo, pericarditis, urinary retention etc.)	5				
Recovered (N = 17)			66.8 (16.4)	9 (52%)	29.5 (13.2)	All discharged
	Chest pain, coronary angiography	3				
	CVA	2				
	Others (CHF, abdominal pain, pneumonia, diarrhea etc.)	12				

### Risk factors for re-admission

Table 3 describes the variables that were found to be associated with re-admission. Principle variables that were found to be associated with re-admission in univariate analysis included older age (HR 1.03 for every year, 95% CI 1.01–1.04, *p* = 0.002); age above 80 years (HR4.93, 95% CI 1.14–21.29, *p* = 0.032; and higher CCI (HR for every point 1.34, 95% CI 1.24–1.44, *p* < 0.0001). Individual comorbidities included in CCI and comorbidities associated with increased risk for re-admission included history of coronary artery disease, CVA, diabetes mellitus, severe kidney disease metastatic disease and a higher systolic blood pressure during the index admission. Solid organ transplantation was also associated with increased risk for re-admission (HR 4.97, 95% CI 2.09–11.83, *p* = 0.0009). A higher lymphocyte counts before discharge from the index admission, and a higher CRP during admission were associated with reduced risk for re-admission Data about inflammatory biomarkers were available for less than 50% of patients, thus their results should be interpreted cautiously.

**Table 3:** Factors associated with re-admission.

Risk factor	Hazard ratio (95% confidence interval)	*p* Value
**Univariate analysis**		
Charlson comorbidity index (HR for every 1 point increase)	1.34 (1.24–1.44)	< 0.0001
Coronary artery disease	2.91 (1.41–5.9)0	0.03
Diabetes mellitus	2.48 (1.40–4.37)	0.0017
Cerebrovascular disease	2.31 (1.10–4.84)	0.025
Metastatic solid tumor	4.68 (2.26–9.69)	<0.0001
Severe kidney disease	3.17 (0.95–10.57)	0.0592
Solid organ transplantation	4.97 (2.09–11.83)	0.0003
Age (HR for every year)	1.03 (1.01–1.04)	0.0002
Age group 80+	4.93 (1.14–21.29)	0.032
Functional status—independent	0.32 (0.16–0.63)	0.0012
Mean systolic blood pressure 48 h before discharge (HR of each measurement unit increase)	1.01 (1.00–1.03)	0.028
CRP on admission (HR of each measurement unit increase)	0.93 (0.88–0.99)	0.022
Ferritin on admission (HR of each measurement unit increase)	0.99 (0.99–0.99)	0.0006
Lymphocytes on discharge (HR for each measurement unit increase)	0.50 (0.25–0.96)	0.04
Fibrinogen on discharge (HR for each measurement unit increase)	0.99 (0.99–1.00)	0.048
Multivariate analysis*		
Solid organ transplantation	3.37 (2.73–7.5)	0.0028
Charlson comorbidity index (HR for every 1 point increase)	1.34 (1.23–1.46)	< 0.0001

*Multivariate analysis included age, gender, admission length of stay, CCI and solid organ transplantation.

In a multivariate analysis (including age, gender, admission length of stay, CCI and solid organ transplantation), solid organ transplantation (HR 3.37, 95% CI 2.73–7.5, *p* = 0.0028) and CCI (HR 1.34 for every point, 95% CI 1.23–1.46, *p* < 0.0001) remained significantly associated with re-admission.

Treatment with remdesivir (53 patients) or dexamethasone (124 patients, including 48 who received concomitant remdesivir), reflecting severity of disease, and SpO2 before discharge (whether measured in room-air or with oxygen supplement via nasal cannula) were not found to be associated with re-admission, as well as all other variables that were examined. All laboratory tests which are now considered as markers for severity of disease, including inflammatory markers, hematological, kidney and liver enzyme tests, were also not found to be associated re-admission.

### Patients who died without re-admission

Ten patients died following discharge from the index admission. Mean age was 85.0 (9.9) years. Eight (80%) needed assistance in basic ADL or had cognitive impairment. The mean CCI was 5.2 ± 2.2. Only one patient was discharged home, all other were discharged to long term care facilities that were adjusted to receive elderly patients with COVID-19. Mean last measured SpO2 before discharge was 94.78 (2.05), two required oxygen supplementation via nasal cannula. Median time from discharge to death was 14.5 (range 2–59) days. All died out of hospital.

## Discussion

Planning discharge is an important part of dealing with the current high burden on medical services owing to COVID-19 outbreak. Knowing the rates and variables involved in predicting re-admission of COVID-19 patients is crucial in order to give clinicians as objective a tool as possible in discharge planning. While the medical management has evolved in the months since COVID-19 outbreak, the re-admission rates have not changed much. The rate of re-admission in our cohort (9.2%) was slightly higher than most other studies that reported ranges from 2.2 to 11.2%^4–7,9,11,12^. There are few possible explanations for the differences between our cohort and those with lower rates of re-admission. In the majority of these studies the follow-up period was significantly shorter (1–4 months). In the study by Jeon et al., with re-admission rated of half compared to ours, only 25% of patients were above 65 years old, while in our cohort more than 50% were above 60 years old^7^. Only few used nationwide databases to obtained information on re-admission to hospitals different from that of the index admission^7,12^. Our results are similar to those reported by Lavery et al., however they reported on same-hospital re-admission^5^. These rates are also similar to those reported for patients hospitalized with acute respiratory illness secondary to seasonal influenza virus, patients hospitalized with community acquired pneumonia, or patients hospitalized in medical and geriatric wards^21,24–27^. They are lower than reported for patients hospitalized because of exacerbation of chronic pulmonary disease or congestive heart failure^25^.

Causes for re-admissions in our study were not related to COVID-19 in the majority of cases, and nearly half of the patients were considered recovered before re-admission. Patients who were re-admitted with COVID-19 related symptoms returned within the first week after discharge. Similar observations were found by others. COVID-19 related symptoms were the cause of re-admission more commonly when patients were re-admitted in the following 10–14 days following discharge^6,10–12,28^.

In our study, median duration of the index hospitalization was 5 days for both groups. Overall length of stay was not found to be a risk factor for re-admission. Two earlier studies have shown association between shorter length of stay during the index hospitalization and higher re-admission rates for patients with COVID-19, although in both studies length of stay was longer^7,11^. Somani et al. have found that patients who were re-admitted had median length of stay of 4.5 days versus 6.7 days, and Jeon et al. found that patients who returned had median length of stay of 9 versus 17 days. Those studies were conducted during the first months of the pandemic when the medical management was different. In a recent report from the CDC a slight borderline association with index admission length of stay was found^5^. Others did not find an association with length of stay^12^. Of interest, for patients hospitalized for non COVID-19 a longer duration of stay is usually associated with increased risk of re-admission^20,21^. This may reflect in part the inexperience of the medical stratification in the face of a new disease for which data are gradually accumulating.

The variables that were found to be associated with increased risk for re-admission in our study were mostly non-modifiable and not relate to COVID-19 severity ^4,8,17,18^. Gender, weight, SpO2, the need for treatment with remdesivir or dexamethasone use and laboratory results (besides lymphocyte count) were not associated with increased risk for re-admission in our cohort. Rather, age and higher CCI score reflecting patients’ comorbidities (diabetes mellitus, chronic kidney disease and cardiovascular diseases) were found to be associated with increased risk for re-admission. Similar findings were reported by others^5–7,11,12^. Somani et al. have found higher prevalence of hypertension and COPD among patients with COVID-19 who were re-admitted within 14 days^11^. Atalla et al. also found that re-admitted patients had a higher burden of comorbidities such as chronic pulmonary disease, liver disease, hypertension, cancer and substance abuse^6^. Both did not find an association with age or gender. In contrast, Jeon et al. and Lavery et al. have found that men and older age, as well as higher CCI, were more frequent among re-admitted patients^5,7^. Hypertension and malignancy were also more common in re-admitted non-severe patients with COVID-19^12^. These variables were also described as risk factors for re-admission among medical and geriatric patients, patients with seasonal influenza of community acquired pneumonia^20,21,24,26,27^.

Mortality rates of solid organ transplant (SOT) recipients with COVID-19 patients varied, with case fatality rates depending on the transplanted organ^29–31^. Factors associated with adverse outcome among SOT are similar to non-SOT patients^29–31^. Currently there is almost no data whether SOT is associated with increased risk for re-admission. In two small case-series with short-term follow-up, re-admission rates were 2.7% (1/37 patients) and 5.5% (1/18 patients)^30,31^. In our cohort, 5/13 (38%) of SOT were re-admitted. Prevalence of organ transplantation in out cohort was 2.4% in the index admission, higher than in other series. It is possible that this larger sub-group enabled us to identify transplantation as a risk factor, overlooked in other studies with smaller numbers of transplant recipients. Whether a longer observation period during hospitalization or closer observation as outpatients in SOT recipients is appropriate requires further research.

Increasing age and various comorbidities such as diabetes mellitus or chronic kidney disease found in our cohort to be associated with re-admission are also associated with increased risk for hospitalization and mortality among patients with COVID-19^8,17,18^. The scores which were developed to predict mortality and outcome among hospitalized patients with COVID-19 based on these parameters don’t answer the question which clinical or laboratory findings need to be met before safely discharging those patients^32^.

An important finding of this study is that oxygen saturation was not a factor associated with an increased rate of re-admission. Patients discharged with supplemental oxygen were not re-admitted at a higher rate compared with those discharged without supplemental oxygen. Thus, use of supplemental oxygen at home in patients with apparently severe, yet stable, disease, is appropriate and under appropriate supervision probably carries a small risk for deterioration.

There are numerous studies that evaluated strategies to reduce early re-admissions^25,33,34^. Some interventions, such as pre-discharge planning and post-discharge follow-up have shown to reduce re-admission rates in medical, especially elderly, patients^33,34^. Guidelines for home-care patients with COVID-19 not requiring hospitalization have been published by the major organizations^35–37^.

In times of limited resources, understanding which patients can be managed at home or at a long-term care facility is an important component of the pandemic management, in order to free up resources for those patients who must be hospitalized. In our cohort, 10 patients, mostly elderly with advanced dementia, died within a median time of two weeks after discharge. We believe plans defining goals of care should be implemented in long term care facilities. Treating physicians should discuss do not resuscitate orders and advanced planning of care with their patients and families, especially elderly patients with multiple co-morbidities, malnutrition or significant cognitive decline. Assimilation of a similar approach has been reported from Canada and France^38,39^.

An important limitation of our study is its’ local nature. However, during the study time period treatment of COVID-19 patients were made in 6 departments (2 ICU) from two separate hospitals with different discharge practice and therefore represent a wide spectrum of patients and various clinical practices and data on re-admission was obtained from a computerized system of electronic medical files that have access to information about hospitalizations nationwide.

Our findings may help in guiding which patients can be discharged early, and perhaps more importantly for which patients’ hospitalization does not significantly affect outcome. Considerations regarding the timing of patient discharge are not uniform. While we cannot predict who will return with deterioration or more severe COVID-19 disease based on our results, it seems that the majority of patients can be discharged home with little risk for re-admission. There is no need for patients to be defined as recovered from COVID-19 prior to discharge if they are able to keep proper isolation after discharge.

## Data Availability

The datasets analyzed during the current study available from the corresponding author on reasonable request.
